# The Estimation of Physical Distances Between Oneself and a Social Robot: Am I as Far From the Robot as It is from Me?

**DOI:** 10.5964/ejop.9519

**Published:** 2023-08-31

**Authors:** Jean-Baptiste Lanfranchi, Sophie Lemonnier

**Affiliations:** 1 Université de Paris Cité and Univ. Gustave Eiffel, LaPEA, Boulogne-Billancourt, France; 2 Université de Lorraine, PErSEUs, Metz, France; Università Cattolica del Sacro Cuore, Milan, Italy

**Keywords:** distance perception, social robot, self-concept, egocentric/allocentric asymmetry effects, human-robot interaction

## Abstract

Research on the perception of interpersonal distance has shown the existence of an asymmetry effect which depends on the reference point of the estimation: the distance from oneself to others can be perceived as longer or shorter than the distance from others to oneself. The mechanism underlying this asymmetric effect is related to the object’s cognitive salience. The self often functions as a habitual reference point and therefore one’s own salience may be higher than that of other objects. In this case, an egocentric asymmetry effect appears with a perceived shorter distance from others to oneself. However, if others are more salient than oneself, then the reverse can happen (allocentric asymmetry effect). The present work investigates if asymmetry in self-other(s) distance perception changes when the other is a social robot. An experiment was conducted with 174 participants who were asked to estimate the distance between themselves and both robotic and human assistants on a schematic map of a hospital emergency room (between-subjects design). With robust ANOVA, the results showed that the participants felt closer to the human assistant than to the robot, notably when the person served as the estimation reference point. Perceived distances to the social robot were not significantly distorted. If a rather allocentric effect with the human assistant might reflect an affiliation goal on the part of the participants, the absence of effect with the social robot forces us to reconsider its humanization. This could nevertheless reflect a purely mechanical and utilitarian conception of it.

In 1985, Codol showed that an asymmetry effect exists in the estimation of interpersonal distance and is dependent on the point of reference of the estimation. In a series of studies on perceived physical distance (“how far are you from others?” versus “how far are others from you?”), participants were asked to estimate either from a graphical depiction or from standing in a room how far they were from the specific others. The findings of these studies showed that the distance of others to the self was consistently underestimated comparatively to the distance from the self to others. For Codol (1985), feeling that our personal space is threatened leads to the activation of a self-centering schema effect, which is the underlying explanation for this egocentric asymmetry. A later study showed that in addition to the management of personal space, the fundamental processes of affirmation and defense of the specificity of personal or social identity are at work (Codol et al., 1989). Asymmetrical distance perception has also been observed in the context of social perception, for example achieving in-group assimilation (self *vs*. others) and maintaining out-group differentiation (us *vs.* them).

Building on findings from these studies, Jarymowicz et al. (2016) argued that this effect of egocentric asymmetry can be explained by a typical cognitive bias extensively studied in social psychology: self-representation has a privileged position as a habitual reference point in social comparison (Codol, 1984; de la Haye & Penvern, 2002; Holyoak & Gordon, 1983; Srull & Gaelick, 1983). Indeed, like other perceptual, spatial, and semantic standards (Rosch, 1975; Sadalla et al., 1980; Tversky, 1977), familiarity with another’s personal qualities, and the number and salience of shared traits are more likely to incite feelings of closeness than the opposite. Nonetheless, this asymmetric effect can also occur allocentrically (i.e., oneself closer to others versus others closer to oneself), notably when dispositional and contextual factors are likely to favor reference to others over ourselves. Deindividuation, anonymity of participants, openness to others and saliency of the stereotype of the other, for example, favor an allocentric rather than an egocentric effect (Jarymowicz et al., 2016; Kamińska-Feldman, 1991).

This brief review of the literature shows that varying the reference point of estimations of interpersonal distance perception can serve as an indirect and heuristic measure of how we conceive ourselves in relation to others. But what becomes of this paradigm when it comes to understanding the relationship between a human being and a social robot? Social robots are autonomous robots designed to interact and communicate with humans. They are increasingly used in a variety of settings, such as health care (Papadopoulos et al., 2020), education (Belpaeme et al., 2018) or home care (Shishehgar et al., 2018). Physically, they can be made to appear machine-like or animal-like, but it has been shown that social robots with human-like features are more effective at facilitating communication with people (Kanda & Ishiguro, 2017).

The proxemics, defined as the human preferences of distance (Hall et al., 1968), is used to evaluate how a human feels about a social robot and thus study the different characteristics of the social robot or the interaction situation that may have an impact. Many studies seem to show that an anthropomorphic design of the robot does not facilitate acceptability (Cornelius & Leidner, 2021), nor the proxemics (Walters et al., 2009), probably due to the uncanny valley effect (Mori et al., 2012). On the other hand, the quality of interactions, such as interactions close to those that one can have with another human, seems to have a positive influence, and decrease the proxemics (Huettenrauch et al., 2006). We can for example cite the work of Walters et al. (2009) who studied the effect of different attributes or factors of robots (e.g., mechanoid robot, humanoid robot, verbal communication, verbal interaction, giving object, passing) on comfortable approach distances. Thus, interactions between social robots and humans have shown tremendous potential in both richness and scope, especially for robots designed to interact in the hospital setting, where they are thought of as tools to help patients, from the pediatric to the geriatric population, even providing emotional support (Prescott & Robillard, 2021).

To our knowledge, no studies to date have been conducted on the asymmetry effect in interpersonal distance perception when one agent is a social robot. However, two aspects of the literature allow us to think that this asymmetry effect in front of a social robot could be observed: 1) in relation to the study of the proxemic effect described above, personal experience with robots in particular seems to diminish a person’s personal space around robots (Takayama & Pantofaru, 2009); 2) the similarity of psychological mechanisms, in particular of social cognition, which is known when interacting with a human and that is also observed when interacting with a robot (Wykowska, 2020).

That being said, two investigations do make use of Codol’s paradigm with non-human stimuli. A study by Housiau (2004), considered estimates of distance perception between oneself and pigeons. The results showed an egocentric effect with humans and an allocentric effect with pigeons. The latter effect appeared to occur irrespective of the status of the human (self or anonymous), meaning that a human is, on average, perceived as being closer to a pigeon than pigeons are to humans. The author largely attributed these results to differences between humans and non-human species (Deconchy, 2000). Another perspective, however, suggests that the self may not be able to play a sufficiently prominent role as an estimation reference point when we consider the stereotypical representation of eight pigeons positioned peacefully on a square. A study by Formanowicz and Karylowski (2011) compared the estimation of distance between oneself, represented by a black dot labeled “yourself”, and a red triangle, without any additional further information regarding placement. The red triangle, in this case, was perceived as being further away from oneself (as reference point) than when the distance to oneself was estimated using the red triangle as the reference. The effect of allocentric asymmetry disappeared, without becoming egocentric, when the visual brightness of the red triangle was diminished by surrounding points of different colored geometric shapes (square, pentagon, cross, etc.). Here, again, the self no longer seems to play its usual role as a reference point when the stimuli (dots) are not labeled as representing humans.

We therefore wondered whether an asymmetry effect would occur in relation to social robots and whether this effect would be amplified by the salience of human-like attributes. The presence of such effects would be encouraging for developing indirect measures of social robot perception alongside questionnaire-based attitude measures (Nomura et al., 2006; Spatola et al., 2021).

In this exploratory perspective, we made two hypotheses:

(H1) - The perception of distances between oneself and a social robot will be asymmetric depending on the estimation reference point.

(H2) - This asymmetry will be more pronounced when the social robot has extended communication capabilities.

Materials, data, detailed results are available through the Open Science Framework (see Supplementary Materials).

## Method

### Participants

The experiment involved 174 participants. Two experimenters coded participants’ responses separately, and after discussion, rejected five that were too illegible or degraded. The 169 selected participants (including 25 men = 15%) were all undergraduate students enrolled at the University of Lorraine in France, without exclusion criteria. Participants signed a consent form and were not compensated. The average age was 21 years (*SD* = 4.3 years).

We did not calculate a required sample size in advance given the exploratory nature of the study. However, a post hoc sensitivity analysis was performed using G*Power Software Version 3.1.9.6 (Faul et al., 2007) to determine the minimum effect size that could be achieved with 169 individuals. The results showed that with a statistical power of .80 and a significance level of α = .05, the minimum effect size to be detected was f = 0.22 (rather moderate-sized effects).

### Material

The material consisted of two sides of an A4 sized sheet of paper (see the ‘Verbatim Instructions.pdf’ in Supplementary Materials). The facing page provided participants with text describing a scene placing the participant in an interactive situation that took place in a hospital waiting room. The situation participants were asked to imagine was that they were waiting in the emergency room for news of the person they had accompanied to seek care for a sprained ankle. Indeed, in order to create a scenario with different possibilities of interaction, we wanted a public place, where everyone can project themselves, and where meeting a social robot is credible in the medium term. We chose the hospital because many studies tend to show that many social robots are already implemented there, as mentioned in the introduction, with very encouraging results (González-González et al., 2021, for a systematic review). The next paragraphs of this text described an interaction with an administrative assistant based on experimental variables we describe in the next section.

**Figure 1:**
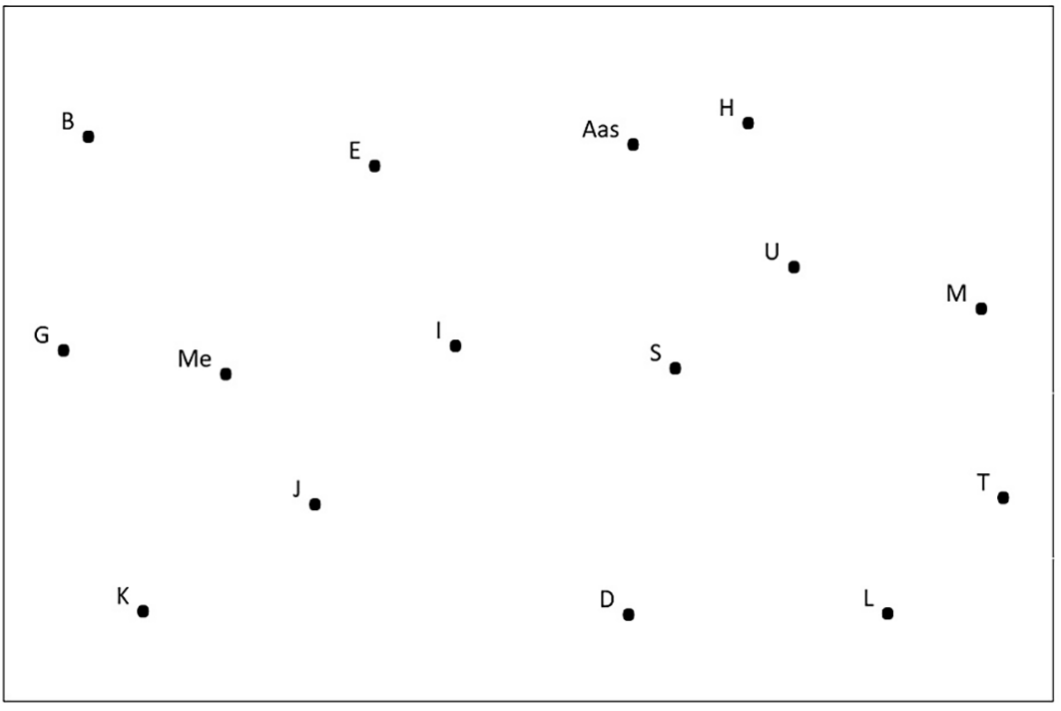
Sample Drawing of the Space with the Administrative Assistant (Aas)

On the back of the A4 sheet was a pictorial representation of the physical situation (map drawing) on which participants were asked to estimate physical distances and respond to several socio-demographic questions. The drawing consisted of 15 points scattered within a frame measuring 16 cm wide by 10.6 cm high. Thirteen points, labeled with letters of the alphabet, represented other people waiting in the emergency waiting room with the participant. The two remaining dots were labeled “Me” for the participant’s position, “Aas” for administrative assistant or “Ras” for robot assistant (see Figure 1). A scale indicated that two centimeters on the drawing of the space represented one meter. The distance between “Me” and “Aas” / “Ras” was 7.4 cm (3.7 meters).

#### Assistant Type (Human vs. Robot)

The second paragraph describing the situation introduced the administrative assistant as being either a person or a robot. In this section, the robot described its capabilities (e.g., “able to understand natural language, provide information and conduct an interview”) and the human assistant described her mission and objectives. On the map drawing, the dot is labeled as either “Aas” (administrative assistant) or “Ras” (robot assistant).

An image of either the robot or the human assistant was provided to the participants (see ‘Human_and_Robot.jpg’ in the Supplementary Materials). The photo of the robot was chosen from those of 19 robots evaluated in a pre-test phase involving 113 students (see ‘Pre-test - Photos of the robots.pdf’ in the Supplementary Materials). For each of the 19 robot photos, the responses were aggregated according to three 7-point Likert scales (Not human at all – Very human; Not realistic at all – Very realistic; Unattractive – Attractive). The selected photo represented REEM-C (from PAL Robotics), an expressionless humanoid robot. Among all the robots presented, this was median in terms of human characteristics attractiveness, with a good score in terms of human likeness and realism.

For the image of the human assistant, we used a random face generator website (Wang, 2019). One judge chose an image of a woman that elicited a neutral response from him: neither strange, such as a significant failure of the AI to generate all the correct parts of the face, nor alarming, with an important asymmetric position of the ears and eyes. Only the absence of negative feedback from two other participating judges validated the final choice. We considered the image of a woman rather than a man, as administrative assistants are most often women in French hospitals (Ministry of Health and Prevention of France, 2022).

#### Interplay (Low vs. High)

The next paragraph introduced the fact that the assistant would soon interact with the participant, the level of which could be *Low* (i.e., distributes a form to be completed), or *High* (i.e., gives a detailed update about the person the participant accompanied and conducts an interview). Consequently, the repertoire of various forms of interaction could differ in terms of richness, depending on the scenario.

#### Reference Point (Me vs. Assistant)

Below the drawing of the space was a question regarding distance estimation, the meaning of which varied. The reference point could be either the assistant (“how far am I from the human assistant/robot?”), or the participant (“how far is the human assistant/robot away from me?”).

### Experimental Design and Procedure

We used a 2 (assistant type: human *vs*. robot) x 2 (interplay: low *vs*. high) x 2 (reference point: me *vs*. assistant) between-subjects experimental design. The participants were randomly allocated to the experimental conditions.

The experimenter began by presenting the framework of the study and instructing participants to refrain from using rulers, objects, or their fingers to estimate distances, but rather to imagine building a mental representation of the scene and estimating distances in meters using the scale provided on the schematic diagram. Completion time averaged 15 minutes and entries were logged anonymously.

### Data Analysis

All analyses were conducted using a dependent variable set to 100: distance estimations in meters proposed by the participants were related to the real distance of the graphical plane. Thus, an estimate of 110 translated an overestimation of 10% of the real distance between oneself and the assistant situated within the plane. These recoded data were analyzed using a three-way robust between-subjects’ ANOVA. We based our decision to use a robust ANOVA on the observation of Q-Q plots, Shapiro-Wilk tests and Levene’s homogeneity of variances test, which revealed on the one hand, a non-normality of the data, significant in six out of eight experimental conditions (*p* < .01), and on the other hand, the trend significant presence of non-homogeneous variances, *F*(7, 161) = 1.91, *p* = .071. Wilcox’s Q test and Cohen’s robust *d* were then applied considering the 20% trimmed means (*M_T_*) and the 20% Winsorized standard deviation (*SD_W_*) as robust estimators (Algina et al., 2005; Wilcox, 2017). The “Walrus” module (Love & Mair, 2018) added to the Jamovi 1.2 software (Jamovi Project, 2020) together with the WRS2 package under R (Mair & Wilcox, 2020; R Core Team, 2019) served as references for all the above analyses.

## Results

**Figure 2:**
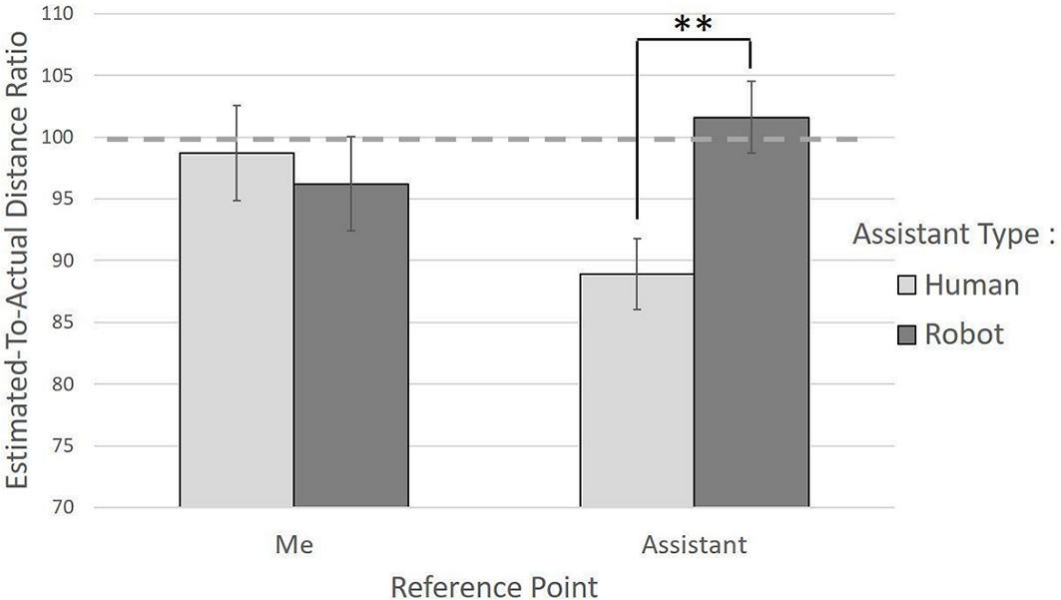
Effect of the Reference Point as a Function of the Type of Assistant on the Estimation of Interpersonal Distances *Note.* A ratio of less than 100 indicates an underestimation of the actual distance.

A main effect of assistant type revealed that the human assistant was perceived closer to the “Me” point (*M_T_* = 93.58, *SD_W_* = 12.22) than the robot (*M_T_* = 98.99, *SD_W_* = 14.57), *Q* = 4.09, *p* = .047, robust Cohen’s *d* = 0.24. Moreover, this effect was expressed differently depending on the reference point of the distance estimation, *Q* = 8.22, *p* = .006 (see Figure 2). No other main effect or interaction was detected as significantly in this study (see ‘Detailed Results.pdf’, Table S2 in the Supplementary Materials).

Within this interaction, one of the six post-hoc comparisons, tested according to Hochberg’s method (Hochberg, 1988), indicated that the human assistant (*M_T_* = 88.86, *SD_W_* = 10.85) versus robot assistant (*M_T_* = 101.56, *SD_W_* = 11.27) was perceived as being much closer to the “Me” point, but only when the reference point of the question asked was the human assistant, *p* =.002, robust Cohen’s *d* = 0.72. On the other hand, the human (*M_T_* = 98.75, *SD_W_* = 15.55) versus robot (*M_T_* = 96.20, *SD_W_* = 15.02) difference was very small and insignificant with “Me” as a reference point, *p* = .64, robust Cohen’s *d* = 0.11.

## Discussion

Our primary objective for this experiment was to test whether the asymmetry effect in the perception of interpersonal distance between oneself and another human being according to the estimation reference point also exists for perceived distances between oneself and a social robot.

While our results tend to underestimate the perceived self–other distances when the other agent is a human assistant, the observed difference is not significant with oneself as the reference point. This underestimation is totally absent when perceived distance estimation is performed with a robot. We note that the participants in this study gave fairly accurate distance estimations when they consider themselves in relation to a robot, regardless of the experimental condition. We also, however, observe a significant difference in perceived distances between the human assistant and the robot when each serves as the estimation reference point, with a tendency to feel closer to the human than to the robot. Finally, our results indicate that enriching relational attributes of either assistant type (human or robot) does not lead to any significant effect in terms of interpersonal distance perception. Therefore, by maintaining sufficient statistical power, the use of robust ANOVA allowed us to detect some effects without necessarily assuming that our observations were normally distributed with homogeneous variances. The use of conventional ANOVA would have led to the absence of significant effects for all the factors of the experiment (see ‘Detailed Results.pdf’, Table S1 and S2 in the Supplementary Materials).

The allocentric appearance effect suggests to us that the salience of the human assistant stereotype tends to outweigh the representation of the self in this context. One could consider that an aim of affiliation, or feeling of likeness with the assistant, contributes to this salience and thus to the distorted perception of distance (Stel & van Koningsbruggen, 2015).

Regarding the social robot, the absence of effect on distance perception seems to imply that participants treat their perceptions of distance in a purely “geometric” manner, suggesting that the robot itself is perceived as purely mechanical, which participants regarded with relative indifference: the self as reference, or activation of salient, fairly rich social features of this robot, appears to have no effect on distance perception between two points.

In order to change the status of the social robot to make it more human, one possible working direction would be to manipulate (augment) its outward expression of ‘emotions’ or ‘desires’ (Haslam et al., 2008). The scenario given to participants in this study, which we designed specifically to help them visualize the robot and immerse themselves actively participating in the situation, focused only on the robot’s skills (e.g., “it is able to understand natural language, provide information and conduct an interview”). Adding a dimension of empathy framed as a soft skill might have helped make it seem more human. An evaluation of the degree of humanization/dehumanization of the robot according to the evolution context must then be considered (Spatola et al., 2021). Indeed, ending the scenario with judgment questions about the robot to make the participants more aware of their own perceptions of the robot, might have made the robot’s attributes even more salient while providing a manipulation check.

Demonstrating that it is possible to provoke asymmetry effects in interpersonal distance perception between oneself and a robot would be an original and resourceful way to gauge how we perceive their position on a continuum between an inhuman (rather the case of our study), animal or human status. In this way, the experimental paradigm proposed by Codol brings a relevant perspective to the ongoing debate on the advantages and disadvantages of humanizing social robots.

While the degree of humanization undeniably increases the quality and quantity of social interactions with a social robot (Spatola, 2019), it is also a source of a social comparison that can lead to identity threats regarding beliefs about human uniqueness and its agency (Giger et al., 2019). Modulating the perceived distance between oneself and a social robot could then be a first indirect measure of these issues, alongside more complex and direct investigations (e.g., attitude scales).

There are several limitations to the results of our experiment. On the one hand, this study was carried out only with a convenience sample made up solely of psychology students with very few men. On the other hand, the scenario, based on a hospital emergency room situation, does not allow us to know what would happen in a much less emotionally charged situation, such as a train station lobby or a shopping center. Finally, regardless of the scenario or the degree of humanization to be reviewed, the physical humanoid aspect of the social robot has not been manipulated here. It would be wise to use the range of social robots already installed in public places to perform this experiment with people who frequent them. This last limitation naturally leads to a study perspective that might have stronger ecological validity. Indeed, the second person neuroscience framework (Schilbach et al., 2013) shows that human social cognition may function differently when we are facing a social agent. Therefore, we may be better able to identify the conditions of activation of the self when estimating distances by considering the social situation as experienced by people interacting with a social robot they already encountered (‘online social cognition’ with engaged people), rather than simply reading a map with an unknown social robot (‘offline social cognition’ with detached people).

Nevertheless, we believe that our results reflect a certain reality, or specific circumstances, where the social robot presented in our scenario might be considered neither as a human nor as a threat, but rather as a functional tool, regardless of the fact that we highlighted some human-like skills.

Admittedly, our representation of social robots is largely influenced by more advanced industrial applications (Piçarra et al., 2016; TNS Opinion & Social, 2012). It is perhaps in this very form, far from any technophobia and close to a dynamic of acceptance of new technological means, that we prepare ourselves to encounter them much more often in our daily lives.

## Supplementary Materials

The supplementary materials provided are data, codebook, and material that support the findings of this study (for access see Index of Supplementary Materials below).



LanfranchiJ.-B.
LemonnierS.
 (2020). The estimation of physical distances between oneself and a social robot: Am I as far from the robot as it is from me?
[Project webpage including data, codebook, materials]. PsychOpen. 10.17605/OSF.IO/ENKQJ
PMC1050819837731753

## Data Availability

The data, codebook, and material used for this study are freely available and can be found in the Supplementary Materials.

## References

[sp1_r1] Lanfranchi J.-B. Lemonnier S. (2020). The estimation of physical distances between oneself and a social robot: Am I as far from the robot as it is from me? [Project webpage including data, codebook, materials]. PsychOpen. 10.17605/OSF.IO/ENKQJ PMC10508198 37731753

